# Impact of Rural vs. Urban Residence on Survival Rates of Patients with Glioblastoma: A Tertiary Care Center Experience

**DOI:** 10.3390/brainsci12091186

**Published:** 2022-09-02

**Authors:** Ali Alwadei, Ibrahim Alnaami, Kawthar Alenazy, Amal Marei, Leenh O. BaHammam, Sameh Nasser, Abdullah Mansour Alswilem, Ahmed Maklad, Shehata F. Shehata, Mohammad Salem Alqahtani, Abdulelah Al-Shahrani, Ali Balbaid

**Affiliations:** 1 Department of Neurosurgery, College of Medicine, Imam Abdulrahman Bin Faisal University, Dammam 34212, Saudi Arabia; 2 Division of Neurosurgery, Department of Surgery, College of Medicine, King Khalid University, Abha 62523, Saudi Arabia; 3 Department of Radiation Oncology, King Fahad Medical City, Riyadh 11525, Saudi Arabia; 4 Department of Clinical Oncology, Ain Shams University, Cairo 11566, Egypt; 5 College of Medicine, Alfaisal University, Riyadh 11533, Saudi Arabia; 6 King Saud Medical City, Riyadh 12746, Saudi Arabia; 7 Department of Clinical Oncology, Sohag University, Sohag 82524, Egypt; 8 Department of Family and Community Medicine, College of Medicine, King Khalid University, Abha 62523, Saudi Arabia

**Keywords:** glioblastoma, glioblastoma survival, radiotherapy in glioblastoma, rural, urban

## Abstract

Purpose: Although the association between residential location and survival in patients with different cancer types has been established, the conclusions are contentious, and the underlying mechanisms remain unknown. Here, we reviewed the impact of residence on the survival of patients with glioblastoma (GBM). Methods: We conducted a retrospective study to compare the impact of rural and urban residence on the survival rates of patients with GBM diagnosed in Riyadh City and outside Riyadh. All patients in this study were treated in a tertiary care hospital, and their survival rates were analyzed in relation to their residence and other related factors, namely radiotherapy timing. Results: Overall, 125 patients were included: 61 from Riyadh City and 64 from outside. The majority of patients in both groups were aged >50 years (*p* = 0.814). There was no statistically significant difference between the groups in the Eastern Cooperative Oncology Group Performance Status (*p* = 0.430), seizure (*p* = 0.858), or initiation timing of radiotherapy (*p* = 0.781). Furthermore, the median survival rate in the Riyadh group versus the other group was 14.4 months and 12.2 months, respectively, with no statistical significance (*p* = 0.187). Conclusions: Our study showed that residential location had no significant effect on GBM prognosis. However, further studies with a larger sample size are required to delineate the other factors of referral within the healthcare system to facilitate the management of these patients within a specific timeframe.

## 1. Introduction

Glioblastoma (GBM) is the most common primary aggressive brain tumor, accounting for 60% of all brain tumors in adults [1]. The global incidence has increased over the last decade, to approximately 10 per 100,000 population [2,3]. Patients with GBM have a poor survival rate, with 37.2% and 5.1% at 1 year and 5 years, respectively [1]. Although GBM has different subgroups, most patients receive identical therapeutic regimens [1,4,5,6]. The current gold standard treatment option is maximum safe surgical resection, in which the maximum extent of resection is associated with improvements in progression-free survival (PFS) and overall survival (OS) [7]. Combined surgical resection and adjuvant chemoradiotherapy showed a significant improvement in median survival rate and PFS with 2.5 months and 1.9 months, respectively, compared to radiotherapy (RT) alone [8]. Some studies have shown that the timing of RT initiation after surgical resection is a key factor in survival [9,10]. Although the association of rural or urban residence with healthcare access has been studied in many different cancers other than GBM, a controversy has been noted regarding the differences in cancer prognosis between these studies [11]. Additionally, the possible contributions of comorbidities and lifestyle factors related to health, and the disparities in cancer outcomes between rural and urban areas have not been completely investigated. Moreover, a recent study reported that the travel distance considered a significant burden on the patients that live in rural areas [11].

We hypothesized that patients who live in urban areas have an easier access to the healthcare system and thus, a better prognosis and OS. The aim of the present study was to evaluate the impact of rural vs. urban residence on the survival of patients with newly diagnosed GBM and other prognostic factors, in a tertiary care setting.

## 2. Materials and Methods

### 2.1. Study Design

A hospital-based case series study included 125 patients with GBM who were treated at the Oncology Department in King Fahad Medical City (KFMC), Riyadh, Saudi Arabia, and which included patients who underwent surgery at KFMC or were referred after being operated on at other hospitals to complete their treatment at the Oncology Department at KFMC, from 2010 to 2018. For this study, rural was defined as any patients who reside outside Riyadh in cities or towns with no oncology centers and need to be referred to an oncology service to carry out chemotherapy and radiotherapy.

This study was conducted in accordance with the principles of the Declaration of Helsinki. The research protocol was assessed and approved by the institutional review board (IRB) of KFMC, Ministry of Health, Saudi Arabia (registration number with KACST, KSA:H-01-R-012). Confidentiality and personal privacy were respected in all participants.

### 2.2. Data Collection

Data were collected from the patients’ records, based on the following clinical features: age, sex, residency (Riyadh City or outside Riyadh), Eastern Cooperative Oncology Group (ECOG) performance status, comorbidities of diabetes mellitus (DM) and hypertension (HTN), presence of seizure, date of first imaging performed, hemisphere (right vs. left), location (lobe), time from first imaging to surgery (days), intervention type (biopsy vs. resection), postoperative residual tumor (yes vs. no), pathology (GBM vs. GBM variant), RT (yes vs. no), RT type (standard vs. hypofractionated), concurrent chemotherapy initiation (yes vs. no), O^6^-methylguanine-DNA methyltransferase (MGMT) status, and time to start radiation therapy (≤6 weeks vs. >6 weeks).

### 2.3. Data Analysis

After data were extracted, they were revised, coded, and fed into the statistical software IBM SPSS version 22 (SPSS, Inc. Chicago, IL, USA). All statistical analyses were performed using two-tailed tests. Statistical significance was set at *p* < 0.05. Descriptive analysis based on frequency and percentage distribution was performed for all variables, including the patients’ demographic data, clinical data, and treatment received. Crosstabulation was used to assess the distribution of different patient-relevant demographic and clinical data according to residence. Relationships were tested using the Pearson chi-square test and exact probability test for small frequency distributions. Survival analysis using a Kaplan–Meier (KM) curve was conducted to compare survival rates among patients according to time to radiation (TTR), with log-rank test to assess significance.

## 3. Results

A total of 125 patients were included: 61 (48.8%) from Riyadh City and 64 (51.2%) from outside Riyadh City. Of the Riyadh City patients, 52.5% were aged ≥ 50 years, compared to 56.3% of the patients from outside Riyadh City, while those aged ≤ 30 years were 16.4% and 12.5%, respectively, with no statistical significance (*p* = 0.814). Male patients were 47 (77%) in the Riyadh group and 46 (71.9%) in the rural group (*p* = 0.508). Twelve (19.7%) patients in the Riyadh group and 12 (18.8%) in the outside Riyadh group were elderly (aged ≥ 65 years) (*p* = 0.896). The ECOG performance status was less than 2 in 35 (64.8%) of patients from Riyadh, compared to 31 (57.4%) of patients from outside Riyadh (*p* = 0.430). A total of 16 (26.2%) patients from Riyadh and 24 (37.5%) patients from outside Riyadh were diabetic (*p* = 0.177). Moreover, HTN was reported in 18 (29.5%) patients from Riyadh and in 16 (25%) patients from outside Riyadh (*p* = 0.571). Sixteen (27.1%) patients from Riyadh City and 18 (28.6%) patients from outside the city had had seizures (*p* = 0.858) (Table 1).

The distribution of the clinical data of the study sample by residence is presented in Table 2. A total of 47.5% of patients from Riyadh and 48.4% of patients from outside Riyadh had right hemisphere tumors, while tumors were bilateral in 11.5% and 12.5% of patients, respectively; *p* = 0.970. In 32.8% of patients from Riyadh and 20.3% of those from outside Riyadh, the affected lobe was the frontal lobe, and 19.7% of Riyadh patients had multifocal tumors, compared to 29.7% of patients from outside Riyadh (*p* = 0.384). Considering the time of surgery in days, none of the Riyadh patients underwent surgery on the same day, whereas 14.1% of those from outside Riyadh underwent surgery on the same day. In total, 31.1% of the patients from Riyadh and 31.3% of those from outside underwent surgery within 1–7 days. Furthermore, 34.4% of Riyadh patients and 29.7% of those from outside underwent surgery after >2 weeks, which was a statistically significant difference (*p* = 0.021). Resection surgery was performed in 86.9% of patients from Riyadh and in 76.6% of those from outside (*p* = 0.137). Residual brain tumors were present in 83.6% of Riyadh patients and 90.6% of those from outside the city (*p* = 0.240). Pathology showed GBM in 91.8% of patients from Riyadh and in 84.4% of patients from outside. Moreover, GBM variants were diagnosed in 8.2% of patients from Riyadh and in 15.6% of patients from outside (*p* = 0.201).

**Figure 1:**
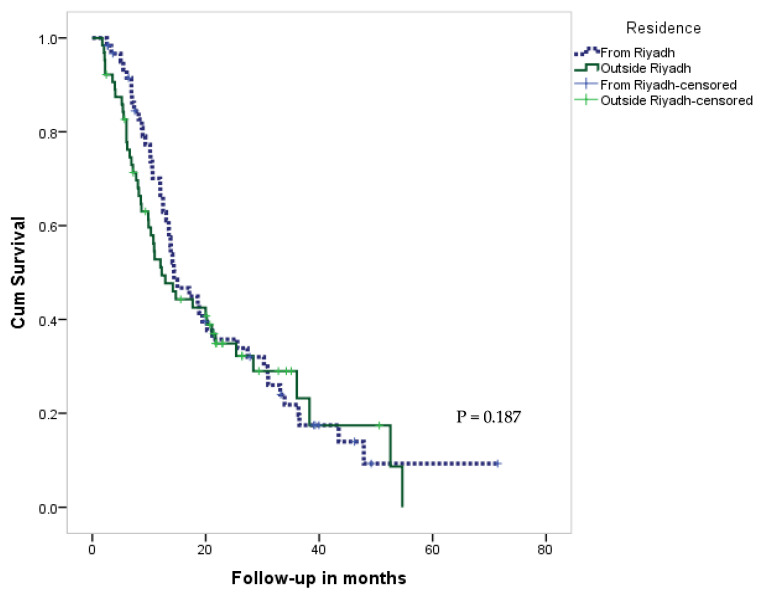
KM curve of survival rate for patients with GBM, by their residence.

The median survival time after diagnosis among patients from Riyadh was 14.4 months compared to 12.2 months for patients from outside Riyadh. In addition, the 5-year survival rate among patients from Riyadh was 9% vs. 0 for the patients from outside Riyadh, with no statistical significance (*p* = 0.187). The overall 5-year survival rate in all patients was 4% (Table 3). The KM curve for the OS rate of patients diagnosed with GBM, according to their residence, is shown in Figure 1. Further data on the survival of the whole study sample are presented in Supplemental Tables S1 and S2.

The distribution of adjuvant therapy according to the patients’ residence is presented in Table 4. Adjuvant chemotherapy was administered to 83.6% of patients from Riyadh and to 70.3% of patients from outside (*p* = 0.078). In terms of RT, 79.7% of patients from Riyadh and 66.7% of those from outside received standard RT, while hypofractionated RT was administered to 20.3% from Riyadh and 33.3% of patients from outside. However, there was no statistical significance regarding the RT type (*p* = 0.110).

**Figure 2:**
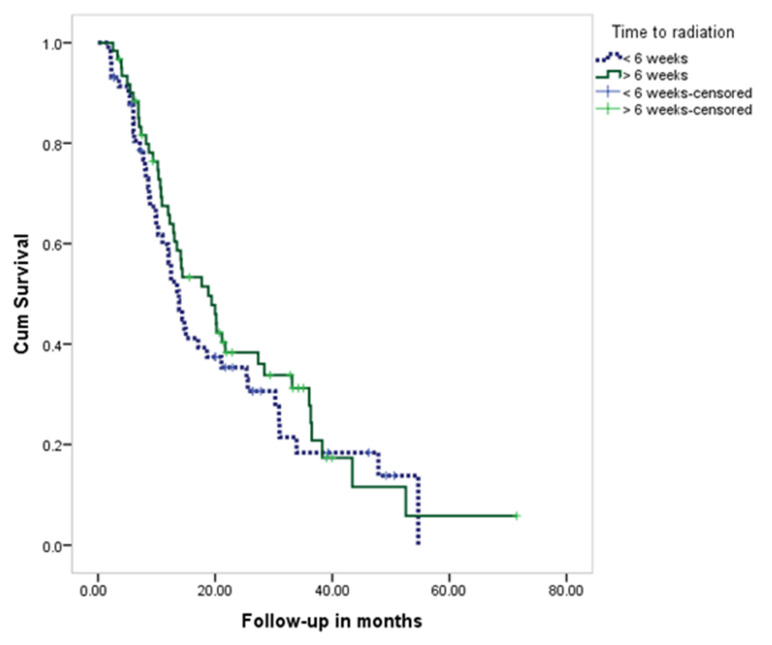
KM curve for survival rate of patients with GBM, according to TTR.

Regarding TTR, it was >6 weeks in 51.3% of all patients (52.5% of Riyadh patients vs. 50% of the outside patients; *p* = 0.781). The total average TTR was 52.9 ± 32.8 days (51.1 ± 20.6 vs. 54.8 ± 41.5 for Riyadh and outside Riyadh, respectively) (Table 5). As for survival rate according to TTR (Figure 2), results showed no significant difference. The median survival time among those who received RT within 6 weeks from surgery was 13.8 months and 18.8 months for those who underwent RT after 6 weeks (*p* = 0.424).

In 6.4% of all patients, MGMT was methylated (3.3% of patients from Riyadh and 9.4% of those from outside). Methylation status of MGMT was unknown in 85.2% of patients from Riyadh and 85.9% of those from outside (*p* = 0.260) (Supplemental Table S3).

## 4. Discussion

GBM is the most common type of glioma, accounting for up to 81% of all primary malignant central nervous system (CNS) tumors in the United States [12]. The geographical distribution and risk factors among patients with GBM have been studied, and they vary between regions. Furthermore, identifiable risk factors that contribute to the overall survival in GBM, such as age, sex, ethnicity, tumor size, and other local tumor characteristics, have been identified. Improved methods for GBM screening and management in high-risk areas may result from a better understanding of regional disparities in glioma incidence and outcomes in the United States [12]. The impact of location of residence has not been studied thoroughly in patients with GBM, whereas in other malignancies, such as prostate, colon, and lung cancers, the association between outcome and location of residence has been established, with lower survival rates among patients living in rural areas [11]. These findings are consistent with those of previous studies. According to a study that examined the impact of travel burden on cancer outcomes, patients in rural areas have worse survival rates and lower quality of life than those in urban areas. Additionally, a considerable distance from the specialist hospital and treatment facility proved to have a negative impact on the stage of cancer at diagnosis and the ability to receive appropriate treatment [11]. Other studies have demonstrated that men in rural areas are likely to have prostate cancer at a later stage due to limited screening systems and prostate-specific antigen testing [11]. Furthermore, patients with colorectal cancer who lived outside major cities in Australia had a lower survival rate. Some of the examined parameters appeared to account for the observed survival disparities. For example, patients in rural locations were less likely to participate in colorectal cancer screening and had less access to oncology services and treatment options, which could have led to lower colorectal cancer survival in rural areas [11]. Moreover, according to a study conducted in the United States, rural patients are less likely to obtain information on cancer therapy trials than urban residents, which may lead to lower survival rates in rural and isolated areas [11].

In terms of the relationship between residential location and cancer stage upon diagnosis, there were disparities between groups. Several studies that examined the differences between rural and urban patients found that rural patients had a more advanced stage of the disease at the time of diagnosis. However, other studies found no relationship between rural residence and late diagnosis [11]. Our study showed no survival differences among patients with GBM who received treatment either in Riyadh or outside Riyadh (*p* = 0.187). However, a trend was noticed in the 5-year survival rate in patients from Riyadh vs. those from outside, with 9% and 0%, respectively, but this is not statistically significant (Table 3). Ostrom et al. reported a higher incidence of primary malignant brain tumors in the northeast of the US than in the central and southern regions [13]. Efird et al. demonstrated the opposite, regarding geographic incidence [14]. Moreover, it is noteworthy that the above statistics represent numerous types of brain tumors and are not a direct reflection of GBM incidence or its geographic variation [12].

Regarding the basic demographic data, which were nearly identical in distribution between both groups, more than half of our population was aged > 50 years in both urban (52.5%) and rural areas (56.3%), while nearly 19% of patients in both groups were elderly (aged ≥65 years). Age did not differ between the two groups (*p* = 0.814) (Table 1). Malay et al., in their study on long-term GBM survival rates, found that younger patients had better long-term survival. However, their national cancer database study of 93,000 patients did not further explore rural and urban relationships and long-term survival rates [15]. Westeel et al. studied the effect of rural residence on lung cancer and reported that overall, patients with lung cancer residing in rural areas have a significantly reduced prognosis compared to that of urban patients [16].

Furthermore, sex, DM, HTN, and the presence of seizures were not significant factors in our study, despite the trends observed for DM and seizures among the patients from outside Riyadh (Table 1). Studies discussing the impact of rural residence and their access to health services in the early 2000s were often significant, as the focus was on providing easier diagnostic and treatment access to that population, which was a challenging milestone. Many studies have found that rural patients have a poorer prognosis and lower long-term survival rates than urban residents [17,18]. In addition, incidence rates of CNS tumors worldwide were studied by Miranda-Filho et al. and reported that South America and former Soviet Union countries had higher incidence of CNS tumors than Japan and the United States [19]. This could be attributed to the fact that urbanization movements came later in South America and the former Soviet Union [19]. In South America, the population in rural areas is slightly higher than in other developed countries, mainly due to poor family planning, which provides a challenge to healthcare access and screening, leading to poorer prognosis in many chronic illnesses and malignancies that usually present late [20].

Since the initiation of the Primary Health Care program in Saudi Arabia, access to healthcare has been satisfactory in both urban and rural areas [21]. The survival rate in GBM has been studied previously in Saudi Arabia, but the effects of rural and urban factors have not been assessed. A single-center study that included 90 patients in Riyadh concluded that ECOG performance status, residual disease, and administration of chemotherapy were significant factors affecting the 5-year survival rate of patients with GBM [22]. In comparison to our study, ECOG performance status, residual disease, and chemotherapy did not differ between the groups, with *p* values of 0.430, 0.240, and 0.078, respectively (Table 1, Table 2 and Table 4). However, the time from diagnosis to surgery in days was significant (*p* = 0.021) (Table 2). Furthermore, a higher trend was observed for same-day surgery among patients from outside Riyadh, which could be related to the initiation of workup and submission of referral data at different institutions, prior to admission at our institution. In contrast, surgical intervention among Riyadh patients took place over the first and second week of diagnosis (Table 2). Similarly, Flanigan et al. reported better survival rates with decreased waiting times after GBM surgery in patients with seizures as the only presenting symptom [23]. In addition, they reported that distance between the patient’s residence and their medical center was not a factor in the waiting time for surgery [23].

The impact of postoperative timing of RT initiation on newly diagnosed GBM is still controversial [24,25]. Some factors may contribute to the increased growth rates of GBM and their radiosensitivity characteristics [24]. Katsigiannis et al. (2019) studied the effect of early initiation of RT and reported that it had no effect on the survival rate of patients [24]. Similarly, other studies have shown that a modest delay might have no negative impact on patient survival [25,26,27,28]. Furthermore, in our study, the initiation of RT postoperatively, <6 weeks and >6 weeks after surgery, did not show a statistically significant difference in survival (*p* = 0.781) between patients (Table 5). However, Alnaami et al. found that a shorter time between surgery and RT initiation is a key factor in improving survival of patients with GBM [10]. Moreover, Sun et al. reported the possibility of worse prognosis in patients receiving RT > 6 weeks post-operatively [9].

The DNA repair protein MGMT is responsible for the elimination of DNA alkyl groups. MGMT expression is associated with an increased response to alkylating chemotherapeutic agents, such as temozolomide. Schaff et al. found a significant association between MGMT methylation and PFS, as well as OS [29]. In our study, MGMT status was examined in approximately 15% of all patients. A lower trend in MGMT methylation was observed in patients from Riyadh (Supplemental Table S3).

The present study has some limitations, including the inherent limitations of retrospective research. Another limiting factor is the lack of MGMT status in most patients. Additionally, the current study analyzed GBM cases over an extended time for which updates in the methods of GBM diagnosis and prognosis have been published

In conclusion, in patients with GBM, the study found no impact of the location of residence on survival. However, although statistically insignificant, the study reveals a trend of improved 5-year survival for patients in urban locations. Further studies are required to delineate other factors within the healthcare system that might delay the diagnosis and treatment of patients with GBM in rural areas, thereby decreasing survival.

## Figures and Tables

**Table 1:** Distribution of study sample characteristics by residence.

Bio-Demographic Data	Residence				*p* Value
	Riyadh		Outside Riyadh		
	No	%	No	%	
**Age in years**					0.814
< 30	10	16.4%	8	12.5%	
30–49	19	31.1%	20	31.3%	
> 50	32	52.5%	36	56.3%	
**Gender**					0.508
Male	47	77.0%	46	71.9%	
Female	14	23.0%	18	28.1%	
**Elderly (> 65 years)**					0.896
Yes	12	19.7%	12	18.8%	
No	49	80.3%	52	81.3%	
**Eastern Cooperative Oncology Group Performance Status**					0.430
<2	35	64.8%	31	57.4%	
>2	19	35.2%	23	42.6%	
**DM**					0.177
Yes	16	26.2%	24	37.5%	
No	45	73.8%	40	62.5%	
**HTN**					0.571
Yes	18	29.5%	16	25.0%	
No	43	70.5%	48	75.0%	
**Seizures**					0.858
Yes	16	27.1%	18	28.6%	
No	43	72.9%	45	71.4%	

*p*: Pearson χ^2^ test.

**Table 2:** Distribution of study sample clinical data by residence.

Clinical Data	Residence				*p* Value
	Riyadh		Outside Riyadh		
	No	%	No	%	
**Brain Hemisphere**					0.970
Bilateral	7	11.5%	8	12.5%	
Left	25	41.0%	25	39.1%	
Right	29	47.5%	31	48.4%	
**Brain Location**					0.384
Cerebellar	1	1.6%	0	0.0%	
Frontal	20	32.8%	13	20.3%	
Multifocal	12	19.7%	19	29.7%	
Occipital	2	3.3%	4	6.3%	
Others	4	6.6%	2	3.1%	
Parietal	10	16.4%	7	10.9%	
Temporal	10	16.4%	16	25.0%	
Thalamic	2	3.3%	3	4.7%	
**Time to surgery in days**					0.021 *
Same day	0	0.0%	9	14.1%	
1–7	19	31.1%	20	31.3%	
8–15	21	34.4%	16	25.0%	
> 15	21	34.4%	19	29.7%	
**Type of surgery**					0.137
Biopsy	8	13.1%	15	23.4%	
Resection	53	86.9%	49	76.6%	
**Residual**					0.240
Yes	51	83.6%	58	90.6%	
No	10	16.4%	6	9.4%	
**Pathology**					0.201
GBM	56	91.8%	54	84.4%	
GBM variant	5	8.2%	10	15.6%	

*p*: Pearson χ^2^ test. * *p* < 0.05.

**Table 3:** Survival rates among patients with GBM, by residence.

Residence	Means and Medians for Survival Time				5-Years Survival Rate	*p* Value
	Mean	SE	Median	SE		
Riyadh	23.7	2.8	14.4	2.0	9.0%	0.187
Outside Riyadh	21.3	2.6	12.2	2.1	0.0%	
Overall	22.4	1.9	14.1	1.9	4.0%	

*p*: Breslow test.

**Table 4:** Distribution of adjuvant therapy to patients with GBM, by residence.

Adjuvant Therapy	Residence				*p* Value
	Riyadh		Outside Riyadh		
	No	%	No	%	
**Radiotherapy received**					0.437
Yes	59	96.7%	60	93.8%	
No	2	3.3%	4	6.3%	
**Radiotherapy type**					0.110
Standard	47	79.7%	40	66.7%	
Hypofractionated	12	20.3%	20	33.3%	
**Adjuvant chemotherapy**					0.078
Yes	51	83.6%	45	70.3%	
No	10	16.4%	19	29.7%	

*p*: Pearson χ^2^ test. *p* < 0.05.

**Table 5:** Time to radiation in all patients, according to residence.

Time to Radiation	Total		Residence				*p* Value
			From Riyadh		Outside Riyadh		
	No	%	No	%	No	%	
≤6 weeks	58	48.7%	28	47.5%	30	50.0%	0.781
>6 weeks	61	51.3%	31	52.5%	30	50.0%	
Mean ± SD (days)	52.9 ± 32.8		51.1± 20.6		54.8 ± 41.5		

*p*: Pearson χ^2^ test.

## Data Availability

The data that support the findings of this study are available from the corresponding author A.A., upon reasonable request.

## Supplementary Materials

The following supporting information can be downloaded at: https://www.mdpi.com/article/10.3390/brainsci12091186/s1, Table S1: Distribution of study sample characteristics by death rate; Table S2: Cox-regression model for predictors of death among the study sample; Table S3: MGMT status in total and by patient’s residence.
