# Supplementary material for: Impact of Rural vs. Urban Residence on Survival Rates of Patients with Glioblastoma: A Tertiary Care Center Experience
Source: Brain Sci. 2022 Sep 2;12(9):1186. doi: 10.3390/brainsci12091186 (PMC9496950; doi:10.3390/brainsci12091186)
Supplement: Supplementary file 1 [file brainsci-12-01186-s001.zip › brainsci-1878751-supplementary.pdf]

Table S1. Distribution of study sample characteristics by death rate.

Bio-demographic data	Death				p-value
	No		Yes		
	No	%	No	%	
**Age in years**					
< 30	10	55.6%	8	44.4%	0.001*
30-49	14	35.9%	25	64.1%	
50+	9	13.2%	59	86.8%	
**Gender**					
Male	30	32.3%	63	67.7%	0.059
Female	3	9.4%	29	90.6%	
**Elderly**					
Yes	2	8.3%	22	91.7%	0.001*
No	31	30.7%	70	69.3%	
**Eastern Cooperative Oncology Group Performance Status**					0.001*
< 2	26	39.4%	40	60.6%	
> 2	4	9.5%	38	90.5%	
**DM**					
Yes	10	25.0%	30	75.0%	0.059
No	23	27.1%	62	72.9%	
**HTN**					
Yes	7	20.6%	27	79.4%	0.127
No	26	28.6%	65	71.4%	
**Seizures**					
Yes	10	29.4%	24	70.6%	0.402
No	23	26.1%	65	73.9%	

P: Log rank test

** P < 0.05 (significant)*

Table S2. Cox-regression model for predictors of death among the study sample

**Factors**	**p-value**	**HR**	**95% CI**	
			**Lower**	**Upper**
Age in years	0.004*	2.35	1.31	4.23
ECOG (>2)	0.017*	3.61	1.26	10.34
Resection surgery	0.045*	6.12	1.04	36.08
Residual	0.007*	0.18	0.05	0.62
Hypofractionated radiotherapy	0.011*	6.93	1.57	30.63
Early adjuvant chemotherapy discontinuation	0.050*	2.28	1.01	5.21

HR: Hazard ratio

CIL Confidence interval

** P < 0.05 (significant)*

Table S3. MGMT status in total and by patient's residence

MGMT	Total		Residence				P value
			From Riyadh		Outside Riyadh		
	No	%	No	%	No	%	
Unknown	107	85.6%	52	85.2%	55	85.9%	0.260
Methylated	8	6.4%	2	3.3%	6	9.4%	
Unmethylated	9	7.2%	6	9.8%	3	4.7%	
Trace	1	0.8%	1	1.6%	0	0.0%	

P: Exact probability test
